# Recent progresses in analytical method development for ^210^Pb in environmental and biological samples

**DOI:** 10.1007/s11356-024-33272-3

**Published:** 2024-04-22

**Authors:** Hong Ren, Xinyu Gong, Lei Zhou, Peng Wang, Yiyao Cao

**Affiliations:** 1 Department of Occupational Health and Radiation Protection, Zhejiang Provincial Center for Disease Control and Prevention, Hangzhou, 310051 China; 2 School of Public Health, Suzhou Medical College, Soochow University, Suzhou, 215123 China

**Keywords:** ^210^Pb, Determination, Environmental, Biological, Review

## Abstract

As a decay product of uranium series, ^210^Pb spreads widely in the nature and imposes strong radiological and chemical toxicity. It is vital to establish reliable and efficient radioanalytical methods for ^210^Pb determination to support environment and food radioactivity monitoring programs. This article critically reviews analytical methods developed for determining ^210^Pb in environmental and biological samples, especially new development in recent years. Techniques applied throughout different analytical steps including sample pretreatment, separation, purification, and detection are summarized and their pros and cons are discussed to provide a holistic overview for ^210^Pb environmental and biological assay.

## Introduction

**Fig. 1:**
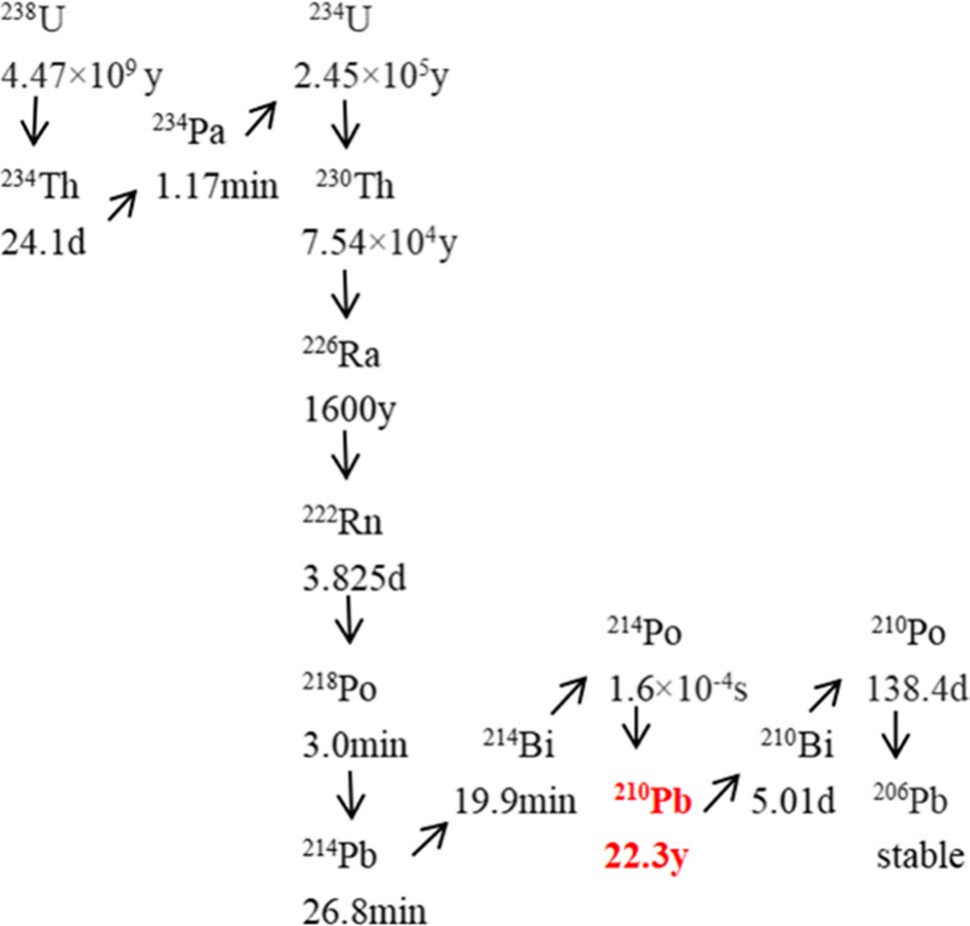
The decay chain of ^238^U (Biggin et al. 2002)

^210^Pb is a radionuclide with 82 protons, 128 neutrons, and a half-life of 22.3 a. It is an important daughter of ^222^Rn in the ^238^U decay series as shown in Fig. 1 (Biggin et al. 2002). ^210^Pb decays into short-lived ^210^Bi (*t*½ = 5 days) by beta decay, and then decays into ^210^Po (*t*½ = 138.5 days) by beta emission. ^210^Pb is considered a highly toxic radionuclide (El Afifi and Borai 2006). It releases low-energy beta particles with energies of 61 keV (19%) and 20 keV (81%) (Blanco et al. 2004; Schayer et al. 2010; Yamamoto et al. 2009), and gamma rays with energies of 46.5 keV.

There are three main sources of ^210^Pb in the environment (Carvalho 1995; Moore et al. 1976): (1) the decay of natural uranium system; (2) the production of nuclear fuel, including mining and smelting of uranium mines; (3) other industrial activities, such as phosphorus mining, phosphate fertilizer production, and coal mining and processing. ^222^Rn and its daughters are important intermediate products in the decay chain of ^238^U, and they are also the main source of ^210^Pb in the atmosphere. During the utilization of minerals, ^210^Pb produced by the decay of ^222^Rn in minerals will be released into the atmosphere. Due to its particle reactive properties, ^210^Pb will be absorbed to sub-micron aerosols and stay in the air for several weeks. The scavenge of ^210^Pb from the atmosphere is mainly through atmospheric convection, sedimentation, rain, and snow, and thereafter, it enters the soil, water system, or deposits on the surface of plants (Kanai 2013; Melieres et al. 2003).

From a radiological point of view, ingestion of radionuclides has a long-term health effect on the human body (UNSCEAR 1993). ^210^Pb in the environment can enter the human body through inhalation, ingestion, or contact through the skin or wound, causing internal radiation exposure hazards (Sha 2004). ^210^Pb that is inhaled into the respiratory tract through the mouth or nose may deposit in various areas of the respiratory system. When ingested, ^210^Pb will enter the gastrointestinal tract through the throat. About 20% of the ingested ^210^Pb can be absorbed and enter into systemic metabolism, which tends to accumulate at a relatively higher extent in specific organs such as the kidney, liver, and bone. ^210^Pb is eliminated from the body through various pathways over time, including feces, sweat, urine, and other channels (e.g., hair, dander), while a small amount of ^210^Pb accumulates in bones with a long biological half-life (Castellino and Aloj 1964; Leggett 1993).

^210^Pb is a bone-seeking radionuclide, and its affinity to bone tissues makes it useful for forensic scientists to estimate post-mortem interval (PMI), which refers to the time that has elapsed since a person or animal has died (Schrag et al. 2012). In addition, ^210^Pb has been widely used as a tracer in sediment dating (Yang et al. 2010), assessment of tobacco and radon exposure (Li et al. 2008; Schayer et al. 2010), soil erosion/disturbance (Matisoff 2014; Porto et al. 2016), and atmospheric, land, and river transport activities (Baskaran 2011; Diaz-Asencio et al. 2017; Teramage et al. 2015).

**Fig. 2:**
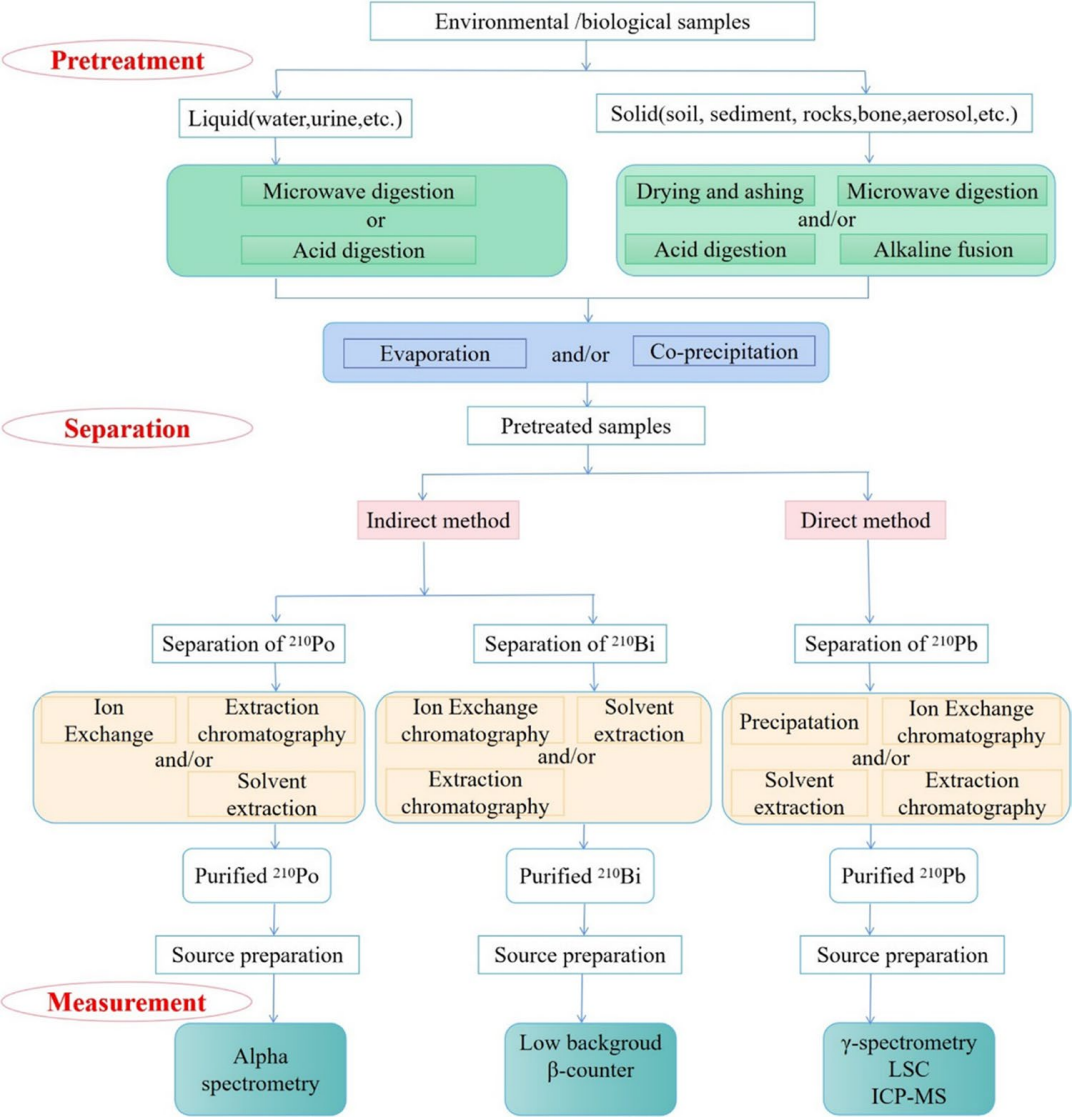
General analytical scheme for determination of ^210^Pb in environmental and biological samples

In short, determination of ^210^Pb in environmental and biological samples is of great significance to protect the environment and human health. However, to the best of our knowledge, there is no systematic review on the methodology development for ^210^Pb environmental and biological assays, as most of the existing reviews are about its application for environmental tracing, dating, and metabolism (Cohen and Howells 1969; Matisoff 2014; Zhang and Xu 2023). This article provides a holistic review of the research progress made for ^210^Pb determination in environmental and biological samples, especially new developments in recent years. The review focuses on analytical techniques applied in different steps, including sample pretreatment, separation and purification, source preparation, and measurement as illustrated in Fig. 2.

In general, the existing methods for the determination of ^210^Pb are classified into direct and indirect measurement methods. The direct method includes counting the low-energy gamma rays (*E*_*γ*_ = 46.5 keV) of ^210^Pb by gamma (*γ*) spectrometry (Barba-Lobo et al. 2021), or measuring the beta activity of ^210^Pb by liquid scintillation counting (LSC) (Stojkovic et al. 2020), or counting ^210^Pb atoms by inductively coupled plasma mass spectrometry (ICP-MS) (Lariviere et al. 2005). The indirect method can be performed through the measurement of its daughter product ^210^Bi with, e.g., gas flow beta (*β*) counter (Franklin et al. 2016) or granddaughter ^210^Po using alpha (*α*) spectrometry (Akozcan 2013). Table 1 complies the progress made in the literature in the past decade regarding analytical methods and their performance for ^210^Pb environmental and biological assays. The subsequent discussion delves into these advancements in detail.

**Table 1:** Analytical methods and performance reported for ^210^Pb determination in environmental and biological samples

Indirect/direct method	Sample	Pretreatment	Separation	Measurement method	Recovery (%)	Detection limit*	Ref
Direct	Dry lake-sediment	Microwave digestion with conc. HNO_3_ + HF	Co-precipitation	LSC (via ^210^Pb)	68–73	11.1 Bq kg^−1^	(Lozano et al. 2010)
	Sludge	Ashing, microwave digestion with conc. HNO_3_ + conc. HCl, evaporation	Sr resin	LSC (via ^210^Pb)	98	12.0 Bq kg^−1^	(Mola et al. 2014)
	Ash, sediment, soil	Drying and homogenization	-	Low-energy gamma spectrometer (via ^210^Pb)	-	8 Bq kg^−1^	(Al-Masri et al. 2014)
	Water	Acid digestion with conc. HNO_3_	Extraction chromatography with U/TEVA™ resin	LSC with optimized *α*/*β* separation (via ^210^Pb)	> 95%	-	(Eikenberg et al. 2014)
	Rainwater, plant	Rainwater: acid digestion Plant: ashing at 420 °C	Rainwater: MnO_2_ co-precipitation	Gamma spectrometry (via ^210^Pb)	-	-	(Yang and Appleby 2016)
	River water, IAEA-447 moss soil	River water: acid digestion with conc. HNO_3_ IAEA-447 moss soil: acid digestion with conc. HCl + conc. HNO_3_ + H_2_O_2_	Sr resin column	LSC (via ^210^Pb)	92 ± 8	1 Bq m^−3^	(Strok et al. 2016)
	Soil, medicinal plant	Soil: sieve Medicinal plant: wet ashing	-	HPGe gamma spectrometer (via ^210^Pb)	-	0.77 Bq kg^−1^	(Chandrashekara and Somashekarappa 2016)
	Beans	Ashing at 450 °C for 48 h Microwave-assisted acid digestion	Sr resin	LSC (via ^210^Pb)	84–84	0.13 Bq kg^−1^ f. w	(Mingote and Nogueira 2016)
	Water	Filter	PS resin	1220 Quantulus LSC	91	-	(Lluch et al. 2016)
	Water	Acid digestion with HNO_3_, co-precipitation with iron hydroxides	Prepackaged Sr resin	LSC (via ^210^Pb)	-	1 Bq m^−3^	(Villa-Alfageme et al. 2016)
	Water	Acid digestion with conc. HNO_3_	Extraction with diammonium hydroxide citrate	3 M Empore™ RAD disk by LSC-PLS (via ^210^Pb)	-	0.02 Bq kg^−1^	(Fons-Castells et al. 2017)
	Bivalve mollusk	-	-	Low-level gamma-ray spectrometry	100	-	(Hurtado-Bermudez et al. 2018)
	Drinking water	Acid digestion with conc. HNO_3_ Iron hydroxide co-precipitation	Sulfate precipitation	LSC X-ray fluorescence	60	0.016 Bq·L^−1^	(Wang et al. 2019)
	Dried shrimp and clam	Microwave digestion system with conc. HCl + conc. HNO_3_ + H_2_O_2_	Sr resin	Ultralow-level LSC (via ^210^Pb)	70	3.85 Bq kg^−1^	(Kong et al. 2021)
	Drinking water	Acidification	MnO_2_ fibers	Low background *γ*-ray spectrometry (via ^210^Pb)	87	0.01 Bq L^−1^	(Aviv et al. 2022)
	Sludge	Microwave digestion with conc. HCl + conc. HNO_3_	PS resin	LSC (via ^210^Pb)	-	5.5 Bq kg^−1^	(Martinez et al. 2023)
Indirect	Mussel	Acid digestion with conc. HNO_3_ + H_2_O_2_	Sr resin, precipitation	Low background gas-flow proportional counter (via ^210^Bi)	46	-	(Rozmaric et al. 2013)
	Bone, liver, and muscle of seabird	Acid digestion with conc. HCl + conc. HNO_3_	Ion-exchange chromatography	Low background gas-flow proportional counter (via ^210^Bi)	-	4 mBq per sample	(Godoy et al. 2014)
	Air	Acid digestion with conc. HNO_3_ + conc. HClO_4_	Anion exchange Spontaneous deposition	Alpha spectrometry (via ^210^Po)	70–80%	-	(Persson and Holm 2014)
	Fish	Acid digestion with HClO_4_ + HNO_3_	Solvent extraction Co-precipitation	Low background *β*-counter (via ^210^Bi)	-	0.2 Bq kg^−1^	(Chen et al. 2016)
	Solid	Acid digestion with conc. HNO_3_ + HF + HClO_4_	Anion-exchange chromatography	*β*-Counter (via ^210^Bi)	91.0 ± 8.3	0.92 Bq kg^−1^	(Jia 2018)
	Macroalgae, seawater	Macroalgae: acid digestion with conc. HCl + conc. HNO_3_ + H_2_O_2_ Seawater: acid digestion with conc. HCl	Macroalgae: ion exchange chromatography (DOWEX 1 × 8) Seawater: MnO_2_ co-precipitation	Alpha spectrometry (via ^210^Po)	-	-	(Uddin et al. 2019)

*f. w. refers to fresh weight. Unless otherwise stated, detection limit for solid samples is expressed based on dry weight

## Sample pretreatment

Because of the low-level concentrations of ^210^Pb in the environment, in most cases, it is difficult to measure it straightforward without sample preparation and obtain accurate results. In order to improve the detection efficiency, the samples need to be homogenized and pre-concentrated to remove the bulk matrix, followed by separation from interferences and purification of the analyte ^210^Pb. Therefore, sample pre-treatment is a key initial step in the ^210^Pb radiochemical analysis process. The specific pre-treatment procedure varies depending on the sample type, with the primary objective to achieve a homogenous sample (mostly in aqueous phase) containing enriched ^210^Pb and eliminated matrix content.

### Pretreatment of solid samples

For environmental solid samples (Bao et al. 2015; Bonotto and Vergotti 2015; Krmar et al. 2014), including soil, sediment, rock, and aerosol, the pretreatment methods used are typically drying and ashing, followed by acid digestion with or without microwave assistance. Lead readily dissolves in warm diluted HNO_3_, while it slowly evolves hydrogen when treated with hot concentrated HCl. Therefore, for effectively leaching ^210^Pb from most soil or sediment samples, a mixture of acids including HNO_3_, HF, HClO_4_, and HCl is widely adopted in various studies (Blanco Rodriguez et al. 2014; Jia and Torri 2007; Sussa et al. 2013). The use of microwave in acid digestion accelerates the speed and completeness for the dissolution of target analyte, allowing for higher sample throughput. For example, Kılıç et al. (2014) utilized a microwave digestion system to pretreat 0.25 g of samples with a mixture of 7 mL of 37% HCl and 3 mL of 65% HNO_3_ when analyzing ^210^Pb in sediments from Golden Horn Bay. The digestion was completed within 35 min.

Because ^210^Pb is highly particle reactive, ^210^Pb can be enriched in aerosols from the atmosphere (Baskaran et al. 1993). To minimize ^210^Pb loss during the analysis of aerosol samples, which are typically collected with silica or glass fiber filters, a fractional ashing method can be employed as a pre-treatment technique in addition to the digestion with concentrated HNO_3_ and HClO_4_ (Persson and Holm 2014). This method effectively prevents the evaporation of lead at temperatures above 500 ℃ as shown in Table 2 (Mao et al. 2018). By adding an appropriate amount of HNO_3_ and HClO_4_ and repeating the operation until complete ashing, this technique ensures thorough sample processing. Additionally, the use of boric acid can help in reducing the ashing time. The advantages of this method are evident in its simplicity, minimal acid consumption, and relatively low laboratory background. However, it may not be suitable for handling a large number of samples (Dai et al. 2015).

**Table 2:** Ashing temperature

Temperature	Time
110 ℃	2 h
200 ℃	2 h
300 ℃	12 h
450 ℃	2 h
500 ℃	24 h

The above-mentioned drying, ashing, and acid digestion approach also applies to pretreat biological samples including bone, lichen, mosses, and food. Differently, specific efforts should be given to the decomposition of organic matters largely contained in biological samples. For this purposes, prolonged ashing, addition of oxidizing reagents such as H_2_O_2_, or the use of microwave digestion became necessary. For example, Wallova et al. (2012) monitored the radioactivity levels of deer bones in Austria, with ashing the bones at 450 °C for 17–22 h in a muffle furnace after slicing. Sert et al. (2011) dissolved lichen and moss samples, which readily absorb ^210^Pb from the atmosphere (Skuterud et al. 2005), with concentrated HNO_3_ and H_2_O_2_, and then treated by concentrated HCl. Kılıç et al. (2014) digested 0.6 g dried mussel sample with 10 mL of concentrated HNO_3_ in a microwave oven at a pressure of 30 bar. To prevent loss of samples and volatile analytes, the sample was placed in cold water immediately after microwave digestion (Henricsson et al. 2011).

Acid digestion method is simple to operate and can effectively decompose organic matter in the sample. However, acid digestion consumes a large amount of acids, and the experiment duration can be quite lengthy, which can be costly and environmental harmful (Huang and Zhu 1981). Microwave digestion technology has many advantages including simple experimental procedure, minimized acid consumption, fast and complete decomposition, and accurate temperature control, which is known as “green chemical reaction technology” (Zhou et al. 2004). However, this technique is not suitable for processing large quantities of samples and complex matrices.

For samples containing refractory fractions, Jia and Torri (2007) applied an alkaline fusion method with fluxes of Na_2_CO_3_ and Na_2_O_2_. Compared with the acid digestion with HNO_3_, HF, HClO_4_, and HCl, the alkaline fusion method delivered comparable results for ^210^Pb. In their study, they applied the alkaline fusion method to various types of solid samples, achieving average chemical yields of 90.0 ± 9.8% for ^210^Po and 88.4 ± 7.1% for ^210^Pb. These results demonstrate the wide applicability and reliability of the alkaline fusion method for solid sample pre-treatment. Compared to acid digestion, alkaline fusion features for much faster accomplishment as it usually operates at higher temperatures (typically 500–1000 °C in alkaline fusion vs. 100–200 °C in acid digestion).

### Pretreatment of liquid samples

Environmental water samples including surface water, seawater, and drinking water are usually pre-concentrated by chemical treatment such as co-precipitation. After collection, the water sample is typically filtered with a filter membrane (0.4–0.45 μm) to remove particles and then acidified to pH 1–2 to avoid the growth of microorganisms. Fe(OH)_3_ or MnO_2_ co-precipitation is often used to pre-concentrate ^210^Pb prior to the subsequent separation and measurement (Kpeglo et al. 2015; Seiler et al. 2011; Yang et al. 2011; Zhong et al. 2020).

For surface water pre-treatment, Burnett et al. (2012) used MnO_2_ co-precipitation to pre-concentrate ^210^Pb. Potassium permanganate and manganese chloride were added to the water sample to generate manganese dioxide precipitation. This approach enabled swift pre-concentration of ^210^Pb from large volume of water samples.

Human urine has a relatively complex matrix with high total dissolved solids and salt content, which needs to be pretreated to reduce matrix effects before chemical separation (Kang et al. 2021). Muikku et al. (2011) determined ^210^Pb activity concentrations in urine by microwave-assisted acid digestion after adding concentrated HNO_3_ and ^209^Po to 90 mL of sample. The use of microwave digestion minimizes the operational time and consumption of reagents and energy (Mingote and Nogueira 2016). However, it cannot completely eliminate the influence of urine matrix; therefore, further separation and purification were followed.

## Separation and purification

Chemical separation and purification are often necessary to remove interferences, and obtain purified and concentrated target analyte. As ^210^Pb can be measured directly for its gamma or beta decay activity, or indirectly via its decay product ^210^Po (*α* emitter) or ^210^Bi (*β* emitter) (see details in the “Measurement” section), therefore, the separation and purification methods were designed accordingly based on the target radionuclide in the detection. For example, interferences to the direct measurement of ^210^Pb beta activity are not only its two progenies but also all other beta emitters and its chemically similar stable elements, such as Ra and Sr. In the cases of indirect measurement through ^210^Po, interferes include all other alpha emitters, such as ^212^Bi, and some ions, such as Fe^3+^and Cr^6+^. And in the indirect measurement through ^210^Bi, interferes include all other high-energy beta emitters, such as ^226^Ra.

Conventional methods for ^210^Pb radiochemical separation include solvent extraction, solid phase extraction, precipitation, or ion exchange chromatography, which are usually applied in combined fashion. However, these separation methods require long time processing (Grate et al. 2020). Over the past decades, highly selective extraction chromatographic materials, such as Sr resin and PS rein (Gimenez et al. 2023; Mingote and Nogueira 2016), have been developed to simplify ^210^Pb separation, and new methods involving a degree of automated separation have also been explored. For example, ^210^Pb was separated from phosphogypsum using an on-line sequential injection (SI) system combined with an ion exchange column (Kim et al. 2008). With the development of flow technology, an advanced lab-on-valve (LOV)—multisyringe flow injection analysis (MSFIA) system was successfully applied to determine ^90^Sr and ^210^Pb (Mola et al. 2014). Flow analysis technology can shorten the analysis time, reduce the consumption of samples and reagents, and thus produce less radioactive waste.

### Precipitation

Lead sulfate (PbSO_4_) precipitation is often used to remove most of the alkaline earth elements. Depending on the solubility, Pb can be separated from Ra(Ba) and Sr in their sulfate/carbonate precipitates (Wang et al. 2019).

As the nitrates of Pb(II) and alkaline earth elements are insoluble in highly concentrated nitric acid (Ostanova et al. 2002), Ra and Pb (precipitate) could be separated from the interfering radionuclides (e.g., U, Th radioisotopes) which are soluble in these media. For example, Lozano et al. (2010) utilized the insoluble properties of Pb(NO_3_)_2_(Ra) in 69% nitric acid medium, and separated Pb(Ra) from U and Th. However, due to the low selectivity of the precipitation method, it is often combined with other separation and purification methods, such as solvent extraction and/or chromatographic separation (Vasile et al. 2016).

### Solvent extraction

Solvent extraction is a method of extracting radionuclides from aqueous phase with organic extractants (Deng and Lin 2022). Solvent extraction can be used to separate ^210^Pb (or ^210^Po, ^210^Bi) from other interfering elements based on their different solubilities in different solvents.

Uddin et al. (2015) used diethyldithiocarbamate diethylamine trichloromethane (DDTC) solution to extract ^210^Po for seawater analysis. Diammonium hydroxide citrate (DHC) also has the potential for lead extraction. Fons-Castells et al. (2017) proposed a procedure to simultaneously extract and measure ^210^Pb, ^228^Ra, and ^226^Ra in drinking water. ^210^Pb was selectively extracted from RAD disk with DHC at a pH of 5.75.

It is reported that ^210^Po can be extracted from HCl solution with tributyl phosphate (TBP) and trioctylamine (TOA) (Younes et al. 2017), or 5% (w/v) trioctylphosphine oxide (TOPO) solution (Grabowski & Bem 2010). ^210^Bi was extracted with xylene with triiso-octylamine (TIOA) in 1 M HCl for air filter analysis, wherein 1 M HCl solution was used for leaching ^210^Bi from the filter (Dlugosz-Lisiecka 2019).

### Chromatographic separation

#### MnO_2_ fiber-column adsorption for ^210^Pb

The adsorption rate of all types of MnO_2_ particles to Pb was reported higher than 85% (Burnett et al. 2012). Aviv et al. (2022) proposed a method for the determination of ^210^Pb in drinking water, in which the water sample was filtered through an acrylic fiber impregnated with MnO_2_. After drying in an oven, the fiber was directly measured by a low background *γ* spectrometer to obtain the activity of ^210^Pb. In the analysis of ^210^Pb in water samples, the combination of ferric hydroxide precipitation and chromatographic separation is also one of the common methods for separating ^210^Pb, and this method has been widely used in combination with various measurement techniques (e.g., LSC, ICP-MS) (Baskaran et al. 2018; Villa-Alfageme et al. 2016).

#### Ion exchange chromatography

Ion exchange chromatography is based on the different affinities of ions and polar molecules onto ion exchangers to separate the target analyte from interfering elements (Zhou et al. 2022). Ion exchange chromatography is one of the common methods for ^210^Pb, ^210^Po, and/or ^210^Bi separation and purification.

**Fig. 3:**
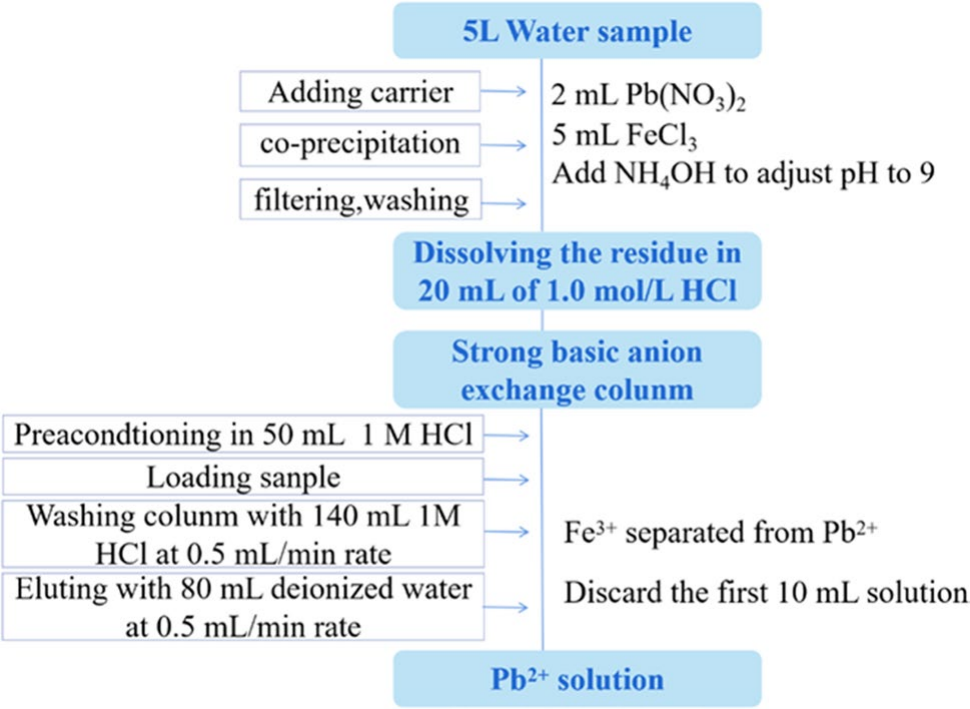
Flow chart of ion exchange chromatographic separation for ^210^Pb in water

Huang et al. (2013) observed that compared with cation exchange resin, anion exchange resin is more effective to separate Pb and Bi from other interferences. The anion exchange resin exhibited the highest adsorption capacity and the best ability to separate impurities in the media of 1.0 mol/L HCl. The detailed separation procedure is outlined in Fig. 3. Dlugosz-Lisiecka and Bem (2012) reported the use of DOWEX anionic reins to separate ^210^Po, ^210^Pb, and ^210^Bi from each other, wherein ^210^Bi radionuclide was eluted by 100 mL of 1.8 M H_2_SO_4_ with an average chemical yield of 80 ± 10%.

Cation exchange resin, such as AG 50W × 8, can separate and purify Po in an acidic environment (< 0.2 M HCl). As the resin contains sulfonic acid functional groups, Po can be eluted first in 0.2 M HCl, followed by Bi in 0.4 M HCl and finally Pb in 2 M HCl. The chemical yield when using AG 50W × 8 cation exchange chromatography was 84.3 ± 0.6% for Pb, 87 ± 1% for Bi, and 92 ± 5% for Po, respectively (Kmak et al. 2017).

#### Extraction chromatography

**Fig. 4:**
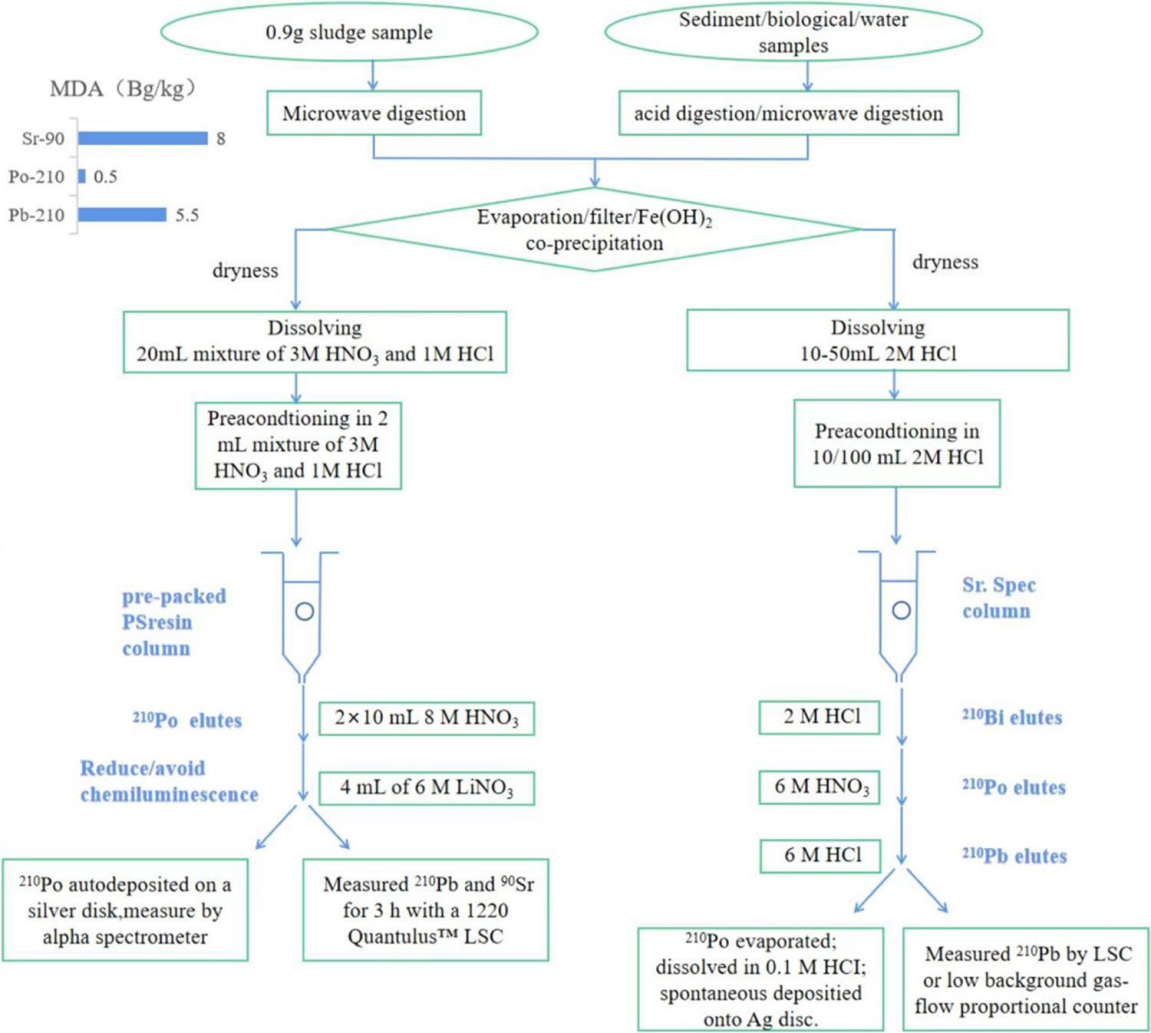
Analytical procedure for ^210^Pb using Sr or PS rein

Sr resin, which consists of 4,4′(5′)-di-t-butylcyclohexano 18-crown-6 in 1-octanol, is a recently developed material for the separation of various inorganic substances based on its different adsorption capacities and selectivity of different target ions (Kong et al. 2021). Since Vajda et al. (1997) proposed the use of Sr resin for the analysis of ^210^Pb and ^210^Po, it has become widely adopted for determination of ^210^Pb and/or ^210^Pb in sediment, biological, and water samples. Polonium can be eluted with 6 M HNO_3_, and lead is eluted with 6 M HCl (Kong et al. 2021; Rozmaric et al. 2013), as outlined in Fig. 4. In addition, DGA resin has also been used to separate and purify ^210^Po in acidic media (e.g., < 1.5 M HCl), eliminating potential alpha emitting interferences (Maxwell et al. 2019).

The recently developed plastic scintillation (PS) resin integrates chemical separation and source preparation into a single step. PS resin is made by fixing a radionuclide selective extractant in a plastic scintillation microsphere, which can be placed in a solid phase extraction (SPE) cartridge. The target radionuclide is retained in the SPE cartridge, and then measured directly by LSC (Gimenez et al. 2023). Martinez et al. (2023) employed PS resin in the analysis of sludge samples collected from drinking water treatment plants. The PS resin selectively retained three radionuclides (^210^Po, ^210^Pb, and ^90^Sr), and effectively separated ^210^Po from ^210^Pb and ^90^Sr, enabling the simultaneous determination of ^210^Pb and ^90^Sr. The procedure, as outlined in Fig. 4, involved loading the digested sample onto the PS resin, from which ^210^Po was eluted with 8 mol/L HNO_3_ separated from ^210^Pb and ^90^Sr solution.

Compared to traditional extraction chromatographic resin (e.g., Sr and DGA), PS resin offers advantages of reduced amounts of reagents and labor required for the analysis, preventing the generation of organic liquid wastes (Bagan et al. 2009, 2012; Barrera et al. 2016).

## Source preparation

### Source preparation for gamma spectrometry

Gamma spectrometry does not require complex chemical separation. For aerosol samples collected with a silica or glass fiber filter, they can be placed in a metal mold and compacted into a known geometry using a hydraulic press for direct gamma spectrometry measurement (Abe et al. 2010). However, direct measurement of ^210^Pb by *γ* spectrometry in water samples without pretreatment is usually not possible due to the low concentrations of ^210^Pb (Aviv et al. 2022). For water samples, co-precipitation with MnO_2_ (Yang and Appleby 2016) as mentioned in the pretreatment section is usually performed prior to the direct measurement of ^210^Pb by *γ* spectrometry. For samples of soil, sediment, ore, etc., the pre-treated samples are packed into suitable containers with known geometry for the *γ* spectrometry measurement (Charro and Pena 2013; Khater and Bakr 2011; Li et al. 2011).

### Source preparation for LSC

The source preparation for ^210^Pb measurement by LSC can be performed following the two approaches: (1) Aqueous samples are counted directly for Cherenkov radiation without the addition of scintillation cocktail. Herein, ^210^Pb is measured indirectly through its daughter ^210^Bi (Stojkovic et al. 2022). (2) Samples are counted after mixing the purified ^210^Pb fraction with cocktail. Prior to mixing with cocktail, ^210^Pb fraction is typically concentrated as precipitation of lead oxalate, lead sulfate, or lead chromate, and then dissolved with diluted acid (e.g., HNO_3_ (Barlas Simsek and Cagatay 2014)) to reduce the quenching effect. Due to the color quenching effect of lead chromate on LSC, it is recommended to precipitate in the form of lead oxalate (Blanco et al. 2004).

### Source preparation for *α* spectrometry

The polonium sources for alpha spectrometry measurement can be prepared using spontaneous deposition or microprecipitation (e.g., CuS (Guerin and Dai 2015) and tellurium (Song et al. 2017) microprecipitation). The spontaneous deposition is the most commonly used, in which polonium is deposited in a metal dish in an acidic environment with stirring and heating in a water bath (80–96 ℃) for 4–6 h (Guerin and Dai 2014; Narayana and Prakash 2010). Polonium can be deposited not only on Ag but also on Ni, stainless steel, and other metal surfaces. Silver proved to be a superior electroplating metal, as it can minimize the loss of polonium during the deposition (Henricsson et al. 2011). In addition, studies have shown that the highest deposition efficiency of Po was achieved when high purity (99.99%) of silver disks was applied (Lee and Chae 2023). The deposition time of Po on the silver disks should not be too long, because studies have shown that when the deposition time exceeded 3 h, measurement of ^210^Po was hindered as the silver disks were covered by oxides and other compounds, resulting in reduced peak resolution of ^210^Po (Szarlowicz 2019).

### Source preparation for ^210^Bi

The primary source preparation method for ^210^Bi involves precipitation. PbSO_4_ precipitate obtained from the chemical separation and purification is left for 5 days on a metal plate to allow for the ingrowth of ^210^Bi. Subsequently, it is counted by gas-flow proportional counter (May et al. 2017). Alternatively, ^210^Bi can be precipitated as Bi_2_S_3_ and filtered, dried, and measured by low background *β* counter (Meli et al. 2011).

## Measurement

To detect ^210^Pb, besides the approach of directly measuring the activity of ^210^Pb, alternative approaches involve deriving the activity concentration of ^210^Pb by detecting its two decay products, ^210^Po or ^210^Bi.

### Indirect method

#### *α* spectrometry (via ^210^Po)

Alpha spectrometry is a detection method to quantify the activity concentration of ^210^Pb by measuring the *α* decay of its granddaughter ^210^Po. After chemical separation, the processed samples are placed in a semiconductor detector to measure the activity of ^210^Po after the spontaneous deposition on a metal surface (e.g., Ag) (Laureano-Perez et al. 2007; Stastna et al. 2010). Kilic et al. (2014) used an alpha spectrometer to measure the activity concentrations of ^210^Po deposited on a silver disk for at least 48 h. When ^209^Po or ^208^Po is used as an internal tracer for ^210^Po, spontaneous deposition may not be able to quantitatively remove all Po, and the residual Po (^209^Po or ^208^Po and ^210^Po) can possibly affect the analytical accuracy. In such cases, it is necessary to remove Po by ion exchange separation, such as with DOWEX (Baskaran 2011; Baskaran et al. 2013). After waiting for 6 months to allow the ingrowth of ^210^Po from ^210^Pb, the samples were re-plated and the activity concentrations of ^210^Pb can be calculated after the second self-deposition. Alpha spectrometry is featured for its low background, high counting efficiency. The main disadvantage of this method is that it takes 6–9 months to allow the ingrowth of ^210^Po from ^210^Pb, so it is not suitable for processing a large number of samples. In addition, polonium is easily adsorbed on the wall of the sample container, so it will suffer losses, resulting in poor repeatability (Cuesta et al. 2022; Vesterbacka and Ikäheimonen 2005). Finally, the recovery rate of Pb could not be evaluated by this method. To determine the recovery rate of ^210^Pb, it can be achieved by measuring stable Pb using ICP-MS (Walsh et al. 2023).

#### *β* counting (via ^210^Bi)

According to “Method for Analysis of Lead-120 in Water” (CNIC 1994) (industry standard EJ/T 859–1994), ^210^Pb was indirectly determined by measuring the radioactivity of its daughter ^210^Bi. Due to the relatively short half-life (5 days) of ^210^Bi, the equilibrium state between ^210^Pb and ^210^Bi is assumed to be reached and maintained throughout the analytical process, from sample collection to the actual measurement. In cases where ^210^Pb and ^210^Bi in the sample do not reach the radioactive equilibrium, the sample is left for about 1 month for the ingrowth of ^210^Bi. *β* counting is a widely used technique for measuring ^210^Bi (Jia 2018; Strok and Smodis 2011). The detection limit of this method is one order of magnitude higher than that of *α* spectrometry method, for example, LOD of ~ 8 mBq by *β* counting vs. 0.03–0.08 mBq by *α* spectrometry (Baskaran 2011).

### Direct method

#### *γ* spectrometry

*γ* spectrometry was first proposed by F. Martinez-Ruiz et al. (2007). It can directly measure the *γ* photon with an energy of 46.5 keV generated by ^210^Pb (Hussain et al. 1996). With improved measurement efficiency of modern *γ* spectrometers, the technology is finding increasing applications in various fields. As it involves simple and non-destructive sample preparation, *γ* spectrometry becomes the first choice for the determination of ^210^Pb in many scenarios. For the analysis of small amount of samples with low activity of ^210^Pb, the well-type HPGe detector is commonly used due to its greater counting efficient in measuring the low-energy gamma rays of ^210^Pb (Sima 2000). However, due to the low *γ* ray energy of ^210^Pb, low branching ratio (4.25%), the self-absorption effect within the sample becomes significant, and in many cases results in insufficient detection limit for environmental and biological applications (Gogrewe et al. 1996; Hussain et al. 1996). Many factors such as sample composition and density need to be calibrated for the full-energy peak efficiency, among which the self-absorption correction for ^210^Pb measurement is particularly important (Hurtado et al. 2007). At present, semi-empirical methods and the Monte Carlo method are the most widely used methods to correct self-absorption effect (Bochud et al. 2006; Iurian et al. 2018).

#### LSC

LSC is a sensitive technique for the measurement of soft beta radiation, allowing for the direct quantification of ^210^Pb (Vajda et al. 1997). It exhibits reduced self-absorption and high counting efficiency. However, this method is sensitive to chemical or color quenching, and it is also necessary to correct the influence of ^210^Bi which requires repeated lengthy measurement (Hou and Roos 2008). The newly developed LSC calibration method by Strok et al. (2016) significantly improved the sensitivity of LSC for determining ^210^Pb in environmental samples, which led to a two-fold increase in detection efficiency. One of the key advantages of this method was its precise evaluation of the detection efficiency of ^210^Bi growth process. This allowed samples to be measured at any time after ^210^Pb radiochemical separation, offering greater flexibility while maintaining a high degree of accuracy.

Vranes et al. (2021) found that 3-methylpyridine-salicylate (3-MPS) increased the efficiency of LSC measurements, suggesting that ionic liquids similar to 3-MPS could replace commercial LSC cocktails. Stojkovic et al. (2022) investigated the effect of various ionic liquids on the detection efficiency of a LSC instrument. Among the tested ionic liquids, they observed that only those containing salicylic acid anions showed a wavelength shift effect, which led to increased detection efficiency.

#### ICP-MS method

It is proposed in the literature (Amr et al. 2010) that the ICP-MS method is a feasible method to detect ^210^Pb. Compared with the above-mentioned radiometric methods, the detection time of ICP-MS is significantly shortened to a few minutes for each sample. However, due to the interferences of ^210^Bi and other polyatomic ions, thorough chemical separation and purification are still necessary. ICP-MS has been used to detect ^210^Pb in a 1 L drinking water sample, and a detection limit of 90 mBqL^−1^ was reported (Lariviere et al. 2005). Due to such high detection limit for ^210^Pb, ICP-MS has rarely been used for ^210^Pb determination in environmental samples with low concentrations of ^210^Pb. Blanchet-Chouinard and Lariviere (2022) proposed a novel procedure based on sequential cloud point extraction (CPE) to reduce detection limits, as CPE could selectively separate/concentrate the analyte, and enable on-line detection. In this study, ^210^Pb isolated from the CPE system was analyzed by ICP-MS with an achieved detection limit of 13 mBqL^−1^ for a 0.35 L water sample. ICP-MS has only recently been applied for ^210^Pb measurement, further improvement in detection limit is still needed.

## Outlook

Technical development for the determination of ^210^Pb in environmental and biological samples, incorporating pretreatment, separation, purification, and measurement, is reviewed in this article, and the advantages and disadvantages of each technique are discussed in detail, as shown in Tables 3 and 4. Both direct and indirect measurement methods have been applied for the determination of ^210^Pb, with each having its own analytical merits. The indirect method through measuring its daughter ^210^Bi or granddaughter ^210^Po takes longer time and requires complicated chemical treatment. The direct method overcomes the limitation of indirect methods that rely on the equilibrium of ^210^Pb and its decay products (^210^Po or ^210^Bi), whereas it still faces challenges related to self-absorption during *γ* spectrometry measurements and quenching effects in LSC. The on-going effort in scintillation cocktail development is useful to further improve the counting efficiency in LSC.

**Table 3:** Advantages and disadvantages of indirect measurement methods

Measurement	Ref	Detection limit	Advantages	Disadvantages
***α* spectrometry**	(Kristensen and Hou 2023)	0.1 mBq/g	Low background count	1. The ingrowth of ^210^Po takes a long time 2. Not suitable for large samples 3. Poor data parallelism 4. Pb recovery could not be determined
	(Fernández et al. 2012)	0.7–16 mBq/L		
***β* counting**	(Jia 2018)	0.92 Bq/kg	The ingrowth time of ^210^Bi and higher detection limit than *α* spectrometry method	Requires chemical treatment
	(Peck and Smith 2000)	4.3 mBq/L

**Table 4:** Advantages and disadvantages of direct measurement methods

Measurement	Ref	Detection limit	Advantages	Disadvantages
**LSC**	(Villa-Alfageme et al. 2016)	0.85 Bq/L	1. Directly measure ^210^Pb weak beta rays 2. Low detection limit	1. The effect of ^210^Bi of new growth on measurement needs to be corrected 2. Chemical and color quenching
	(Pan et al. 2018)	38.0 mBq/g		
***γ* spectrometry**	(Meli et al. 2013)	18.9 Bq/kg	1. Non-destructive measurement 2. No or simple sample pretreatment	1. Low detection efficiency 2. High self-absorption 3. Demand for large sample quantity
	(Grabowski et al. 2010)	< 6.2 mBq/dm^3^		
**ICP-MS**	(Amr et al. 2010)	0.698 Bq/mL	Shorter measurement time	1. Higher detection limit 2. Expensive instrument
	(Baskaran et al. 2018)	45–50 mBq/L		

When the activity concentration of ^210^Pb in a sample is low, the sample needs to be chemically separated, and the activity concentration of ^210^Pb is typically determined through its daughter ^210^Bi or its granddaughter ^210^Po. However, this method is time-consuming. In situations where the ^210^Pb concentration needs to be measured within a short time, direct measurement of ^210^Pb by gamma spectrometry or liquid scintillation counting is a method of choice. In addition, ICP-MS can be selected depending on the experimental conditions to achieve rapid screening.

Considering the impact on the environment and the concept of sustainable development, in the process of chemical analysis, sample preparation is considered to be the main source of pollution (Cerutti et al. 2019; Lopez-Lorente et al. 2022). The concept of green sample preparation and green analytical chemistry is the main trend of analysis today. They all achieve sustainability by reducing the amounts of pollutants in the analysis process (Lopez-Lorente et al. 2022). Therefore, bearing this in mind, when acid digestion is used for sample pretreatment, microwave-induced digestion may be considered the first option to reduce the acid consumption and save energy (Bizzi et al. 2017). For separation and purification, solid phase microextraction (SPE) can be regarded as a solvent-free extraction technology, which provides possibilities for green environmental protection. Besides, it requires shorter processing time and simpler operation compared with conventional separation methods (Risticevic et al. 2009; Wang et al. 2011). Moreover, in recent years, the focus of research has shifted towards designing and developing highly selective new materials that can facilitate the efficient separation and enrichment of ^210^Pb.

With the new development of artificial intelligent (AI), the application automation in radiochemical analysis will undoubtedly reduce reagent consumption and labor intensity, as well as improve sample throughput and operational safety. By far, many automated methods have been developed, but mostly coupled offline with the measurement. Collaborations between material science and other disciplines will likely play a crucial role in shaping the future of ^210^Pb methodologies. We foresee improvement on this basis and integrate it into the analysis process to achieve online chemical separation and measurement.

## Data Availability

Not applicable—no primary data was generated for this manuscript.
